# Rapid and effective response of the R222Q SCN5A to quinidine treatment in a patient with Purkinje-related ventricular arrhythmia and familial dilated cardiomyopathy: a case report

**DOI:** 10.1186/s12881-018-0599-4

**Published:** 2018-06-05

**Authors:** Joanna Zakrzewska-Koperska, Maria Franaszczyk, Zofia Bilińska, Grażyna Truszkowska, Małgorzata Karczmarz, Łukasz Szumowski, Tomasz Zieliński, Rafał Płoski, Maria Bilińska

**Affiliations:** 1 Department of Arrhythmia, Institute of Cardiology, ul. Alpejska 42, 04-628 Warszawa, Poland; 2 Molecular Biology Laboratory, Department of Medical Biology, Institute of Cardiology, ul. Alpejska 42, 04-628 Warszawa, Poland; 3 Unit for Screening Studies in Inherited Cardiovascular Diseases, Institute of Cardiology, ul. Alpejska 42, 04-628 Warszawa, Poland; 4 Department of Heart Failure and Transplantology, Institute of Cardiology, ul. Alpejska 42, 04-628 Warszawa, Poland; 5 Department of Medical Genetics, Medical University of Warsaw, ul. Pawinskiego 3c, 02-106 Warszawa, Poland

**Keywords:** *SCN5A*, Na_v_1.5, Dilated cardiomyopathy, Multifocal ectopic Purkinje-related premature contractions

## Abstract

**Background:**

Mutations of the *SCN5A* gene are reported in 2-4% of patients with dilated cardiomyopathy (DCM). In such cases, DCM is associated with different rhythm disturbances such as the multifocal ectopic Purkinje-related premature contractions and atrial fibrillation. Arrhythmia often occurs at a young age and is the first symptom of heart disease.

**Case presentation:**

We present the case of 55-year old male with a 30-year history of heart failure (HF) in the course of familial DCM and complex ventricular tachyarrhythmias, which constituted 50-80% of the whole rhythm. The patient was qualified for heart transplantation because of the increasing symptoms of HF. We revealed the heterozygotic R222Q mutation in *SCN5A* by means of whole exome sequencing. After the quinidine treatment, a rapid and significant reduction of ventricular tachyarrhythmias and an improvement in the myocardial function were observed and this effect remained constant in the 2.5-year follow-up. This effect was observed even in the presence of concomitant coronary artery disease.

**Conclusions:**

Patients with familial DCM and Purkinje-related ventricular arrhythmias should be offered genetic screening. The quinidine treatment for the *SCN5A* R222Q mutation can be life saving for patients.

## Background

Mutations of the *SCN5A* gene, which encodes the cardiac sodium channel alpha subunit (Na_v_1.5) are reported in 2-4% of patients with dilated cardiomyopathy (DCM) [1, 2]. Mutations in the *SCN5A* gene have been reported in the congenital long QT syndrome and in the Brugada syndrome [3]. A few different mutations of voltage sensors Na_v_1.5 in DCM were characterised (missense: T220I, R219H, R222Q, R225W, E446K, R814W, D1275N, V1279I, D1595H, F1520 L, and I1835T) [2, 4–6]. Most of them are localised to the S3 and S4 transmembrane segments, supporting the hypothesis outlining the dysfunction of the Na_v_1.5 in the pathogenesis of DCM. However, the pathological mechanism of the Na_v_1.5 mutation-induced DCM is not completely understood. The phenotype is associated with different rhythm disturbances, such as atrial fibrillation (AF), the sick sinus syndrome, multifocal ectopic Purkinje-related premature contractions (MEPPCs), progressive cardiac conduction system disease and familial DCM. Arrhythmia (AF, multifocal premature ventricular contractions - MPVCs) often occurs at a young age and is the first symptom of heart disease [4–7].

## Case presentation

**Fig. 1:**
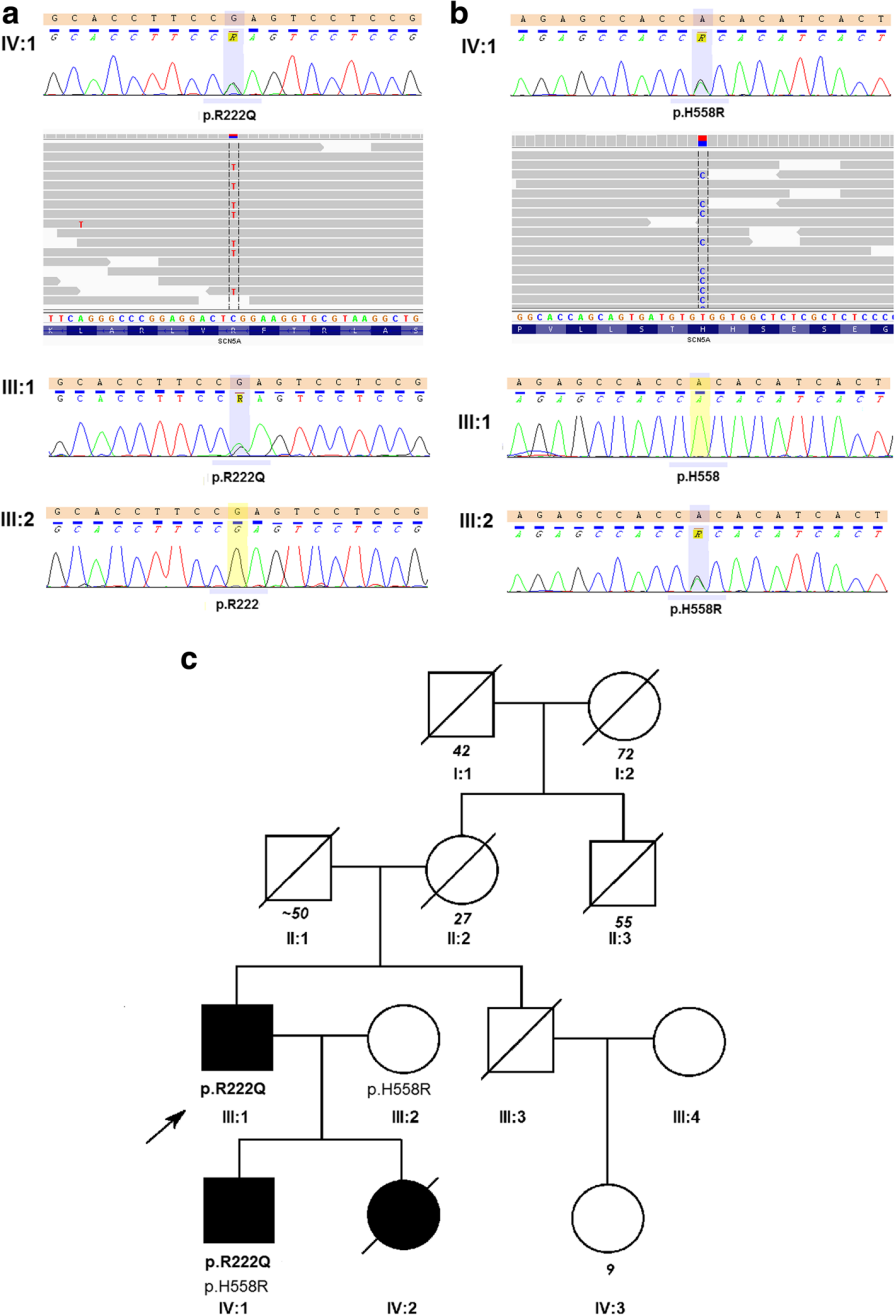
Genetic characteristics of the studied family. Chromatograms and IGV views of *SCN5A* NM_198056.2:c.665G > A (p.R222Q) (**a**) and *SCN5A* NM_198056.2 :c.1673A > G (p.H558R) (**b**), variants and family pedigree (**c**). Pedigree: squares represent males and circles represent females. An arrowhead denotes the proband. A diagonal line marks the deceased individuals. Open symbols denote unaffected individuals

We present the case of a 55-year old male (Fig. 1c - III:1) with a 30-year history of heart failure in the course of familial DCM and MEPPCs. The patient’s family phenotype was characterised in Table 1 and Fig. 1c.

**Table 1:** Clinical characteristics of living affected family members

Subject	III:1	IV:1
Mutation *SCN5A*	p.R222Q	p.R222Q, p.H558R
Age at onset/now (years)	25/57	7/31
Sex	Male	Male
Symptoms	Palpitations, presyncope	Palpitations/SCD **-**successfully resuscitated
NYHA functional class	III	IV**-** > 23 year old **-** HTx
LVdD (mm)/LVEF (%)	68/20	84/10-15
Arrhythmia	MPVCs/nsVT/IVR	MPVCs/nsVT/VT/VF/AF
Conduction disorders	AVB I^o^, RBBB	No
ICD	Yes	Yes /explantation after HTx
Amiodarone	Yes **-** > hyperthyroidism	Yes **-** > unsuccessful
Quinidine treatment		
Quinidine	Yes	No
Symptoms before/after	Yes/no	**–**
NYHA functional class	III- > II	**–**
LVdD (mm) before/after	68/62	**–**
LVEF (%) before/after	20/35	**–**
Arrhythmia during 24 h before/after	65,000 MPVCs/ ~ 4000 MPVCs	**–**

*SCD* sudden cardiac death, *HTx* heart transplantation, *AVB I*^*o*^ first degree of atrio–ventricular block, *RBBB* right bundle branch block, *IVR* idioventricular rhythm, *MPVCs* multifocal premature ventricular contractions, *LVdD* left ventricle enddiastolic diameter, *LVEF* left ventricle ejection fraction, *VT* ventricular tachycardia, *ns* non-sustained, *VF* ventricular fibrillation, *AF* atrial fibrillation, *ICD* implantable cardioverter–defibrillator

**Fig. 2:**
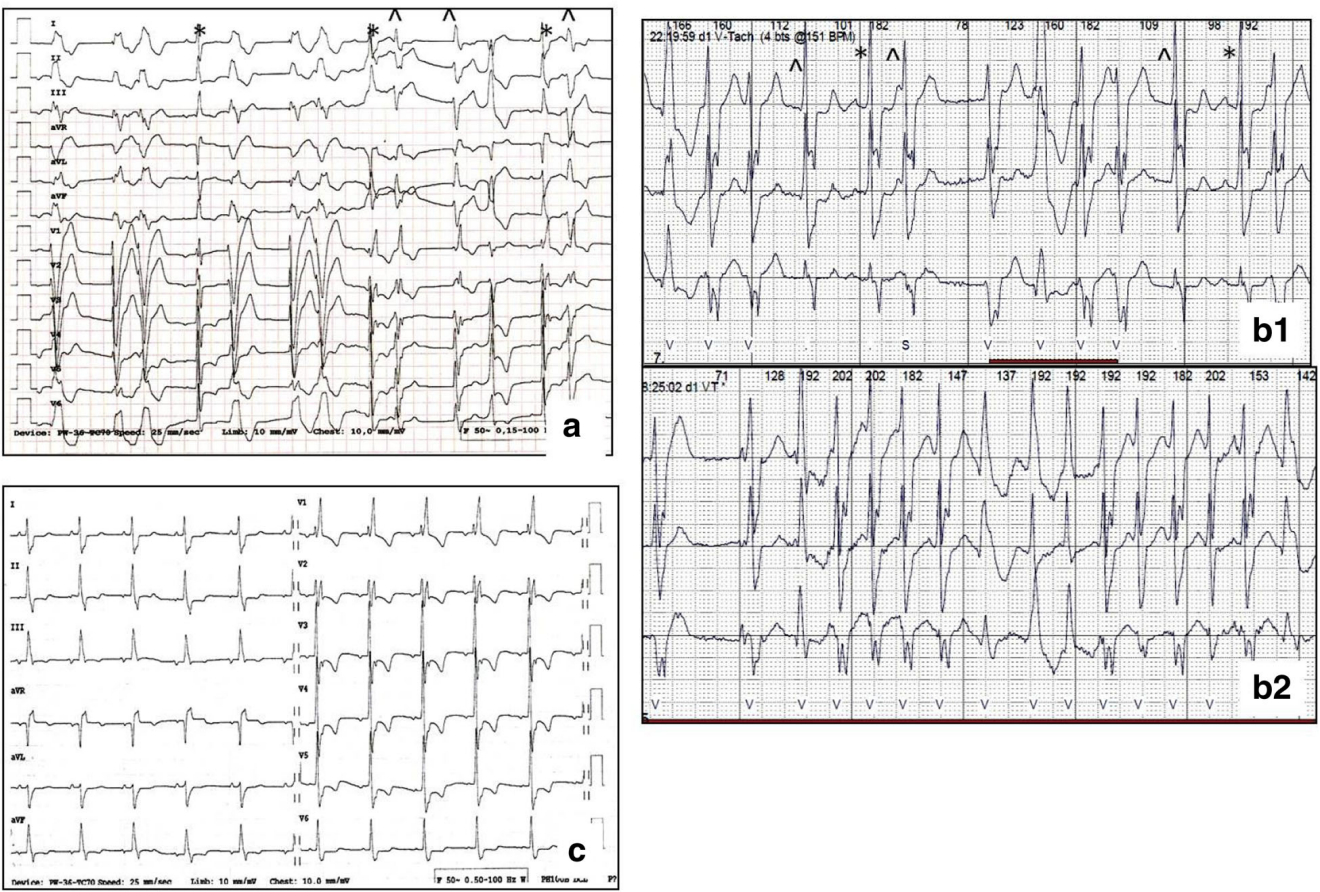
Electrocardiograms before (**a**, **b**) and after (**c**) quinidine treatment. **a** representative 12-lead ECG before quinidine treatment (25 mm/sec., 10 mm/mV), sinus beats (*) with different RBBB pattern, supraventricular and junctional beats (^) with RBBB+ LAH pattern, LBBB-like pattern ventricular extrabeats with variable saxis. **b1, 2** 24-h Holter ECG rhythm strip showed single sinus (*) and supraventricular (^) extrabeats and MPVCs with narrow QRS and different axis (upper panel) and nsVT (lower panel). **c** 12-lead ECG (25 mm/sec., 10 mm/mV) after quinidine treatment - morphology of P differed from sinus rhythm, RBBB with right axis, without ventricular arrhythmia. RBBB – right bundle branch block, LBBB - left bundle branch block, LAH – left anterior hemiblock, MPVCs - multifocal premature ventricular contractions, nsVT - non-sustained ventricular tachycardia

His mother (Fig. 1c - II:2) suffered from unrecognised heart disease with ‘heart palpitations’ and died suddenly at the age of 27; his daughter (Fig. 1c - IV:2) died of heart failure at age of 9, while waiting for a heart transplantation, and his son (Fig. 1c - IV:1) received a heart transplant at age of 23, while experiencing heart failure symptoms along with frequent complex arrhythmia since the age of 20. In our patient, the amiodarone treatment was ineffective and was discontinued after amiodarone induced hyperthyroidism. At 46 years of age the patient had a cardioverter-defibrillator (implantable cardioverter-defibrillator - ICD) implanted. At 49 years of age lead-dependent infective endocarditis (Metyciline resistant *S. aureus* - MSSA) was diagnosed. A cardiosurgical lead extraction, tricuspid valve annoloplasty and epicardial ICD implantation was performed. At 51 years of age, the patient was diagnosed with coronary artery disease (CAD) based on the coronary angiography, which showed permanent occlusion of the LAD distal section with a permanent lack of perfusion in the middle and apical segments of the anterior wall and septum as demonstrated by the SPECT Tc-99 m study. During the next few years the patient presented with NYHA class II symptoms and moderate left ventricle (LV) function impairment (left ventricle ejection fraction - LVEF 40%). At 53 years of age, an increasing number of MPVCs was found to be associated with the worsening of the myocardial function (LVEF 20%). His 12-lead surface electrocardiogram (ECG) showed single sinus (or similar to sinus) beats, with different RBBB pattern and multiple LBBB-like pattern ventricular extrabeats with variable axis (Fig. 2a). The 24-h Holter ECG revealed sinus bradycardia, junctional ectopies, very frequent MPVCs (~ 65,000/24 h) with multiple different quite narrow morphologies, mainly RBBB- and LBBB-like patterns, as well as about 3500 episodes of non-sustained ventricular tachycardia (nsVT) - max. 30 beats, 170/min. and slow ventricular rhythms (4400 episodes) as a consequence of sinus dysfunction (Fig. 2b). The MPVCs comprised of > 80% of the whole-day rhythm.

Due to a lack of therapeutic options, the patient was enlisted for heart transplantation (HTx) in the planned mode. In the meantime, it was decided to perform whole-exome sequencing in the most severely affected living person in the family (the patient’s son). Based on the result of the identification of the known mutation in Na_v_1.5 in both affecteds (the patient and his son), we applied the known antiarrhythmic treatment that led to removing the patient from the waiting list for HTx. In addition, we tried to clarify the differences in the course of the disease between two living affecteds in the family. The clinical characteristics of the proband and his son were summarised in Table 1.

### Genetic testing

The DNA was extracted from the peripheral blood by means of a phenol extraction. Whole-exome sequencing (WES) was performed in the proband’s son (Fig. 1c - IV:1) on HiSeq 1500 using SureSellect Kit (Agilent Technologies) as described previously [8]. In WES, the mean coverage of the DNA sample was 75-fold with 98.6% of the targeted exons covered at least 10× and 94.6% covered at least 20×. The *SCN5A* variants identified with WES were followed-up in the proband’s family with Sanger sequencing using a 3500xL Genetic Analyzer (Applied Biosystems, Foster City, CA, USA) according to the manufacturer’s instructions. The results were analysed using the Variant Reporter 1.1 Software (Applied Biosystems).

The patient and his son (Fig. 1c, III:1, IV:1) carried a rare heterozygous *SCN5A* NM_198056.2:c.665G > A (p.R222Q) variant in the transmembrane helical segment S4, which is the voltage-sensor (Fig. 1a, b). In the patient’s son (Fig. 1c - IV:1), the *SCN5A* NM_198056.2:c.1673A > G (p.H558R) variant was furthermore found, inherited from the unaffected mother (Fig. 1c - III:2).

### Quinidine treatment

The patient was treated according to the ESC/AHA guidelines for treatment of heart failure [9], with optimal dosage of ACEI (ramipril 10 mg/day), beta-blocker (bisoprolol 10 mg/day), diuretic (furosemide 80 mg/day) and spironolactone (25 mg/day) as well as oral anticoagulant (acenocoumarol). Recently, he was also treated with ASA and statin (atorvastatin 20 mg/day) for CAD. Based on the published data [10], quinidine (Quinidine sulphate in tablets a 200 mg) was administered. After the first quinidine dosage we observed a rapid antiarrhythmic effect (Fig. 2c), which was confirmed in the 24-h ECG monitoring (reduction of MPVCs from > 80%/day, including VT/nsVT to 3-5%/day, without VT/nsVT). The echocardiographic examinations repeated over 2 years revealed the sustained improvement of the LV contractile function (LVEF 20-25% → 30-34%). We have observed a significant reduction in heart failure symptoms and a significant improvement in physical efficiency (Table 1).

After 12 months of treatment, the patient presented a mild manifestation of drug-induced diarrhoea and mucous membrane dryness, and had to stop taking quinidine. The lack of compliance resulted in an increasing number of ventricular arrhythmia (MPVCs > 80% in 24-h ECG registration). We have modified the daily quinidine dosage with positive antiarrhythmic response. After the 2 year follow-up we did not find an increasing number of MPVCs. In consequence, the number of MPVCs in the 24-h ECG monitoring was stable (~ 4000/day) and the patient’s myocardial performance remained stable (LVEF-35%).

## Disscusion and conclusions

### SCN5A R222Q variant

*SCN5A* R222Q affects the positively charged arginine which lies on the S4 segment of the DI domain (DI/S4) - one of the four sodium channel voltage sensors of the primary sodium channel in the heart - Na_v_1.5 [11]. This variant was previously described in at least 8 families with DCM/arrhythmias/LQTS and segregated with disease among at least 38 relatives [4, 10, 12–16]. The effect of the R222Q mutation on the Na_v_1.5 function was experimentally investigated [10, 14]. The *SCN5A* R222Q varaint leads to a gain in the function of the mutated channel, which expresses many kinetic disturbances, such as an opening at more negative potentials. Mann et al. [14] suggest that the increasing and rate dependent automaticity of the Purkinje fibre cell is the reason for the MPVCs. It is consistent with a previous study [10], which described the mechanism of MPVCs triggered by an incomplete Purkinje fibre repolarisation and their propagation into the ventricles. In patients with the *SCN5A* R222Q mutation, the entire Purkinje system is changed and can be a source of a variety of ectopic foci, resulting in a phenotype called the MEPPCs syndrome. DCM caused by the *SCN5A* R222Q mutation, seems to be a secondary finding to ventricular arrhythmia, of note we have not observed myocardial muscle thinning in the course of the disease, like in our patient (LV wall thickness was 12-13 mm).

However, the mechanisms of left ventricular dilation in individuals carrying the *SCN5A* R222Q mutation are not completely defined. The patient’s son, in whom we performed the WES study, also carried the well-known *SCN5A* H558R polymorphism, which was previously described in patients carrying the *SCN5A* R222Q variant [12]. The altered current Na^+^ activity may significantly deteriorate the left ventricular function along with the effects of MEPPCs or the presence of atrial fibrillation [17]. In Mann’s study, the R222Q variant of *SCN5A* presented a different expression related to sex, with more genotype-positive males (7 of 10) affected with DCM than females (1 of 7) [14]. That fact was explained as a protective role associated with a higher heart rate in women due to a reduced Purkinje cell excitability.

We observed a significant difference in the course of HF and in terms of age at the onset of symptoms between the described patient (Fig. 1 – III:1) and his children. We only found that the *SCN5A* H558R polymorphism, present in the proband’s son, was inherited from the unaffected mother. However its presence did not exacerbate the course of the disease in the study by Cheng et al. [12].

### Quinidine treatment

The phenotype caused by the *SCN5A* R222Q mutation (DCM, MEPPCs) was found to be responsive to sodium channel blockers [10, 14]. Laurent et al. first reported the association of MEPPCs and left ventricular dysfunction in 3 unrelated families with the *SCN5A* R222Q mutation, stressing that DCM is a secondary consequence of the mutation. The authors found spectacular effect of the quinidine treatment in 2 patients with the arrhythmic phenotype within one family in whom the normalisation of the left ventricular function was noted and in two affecteds from another family who had a normal left ventricular function [10] . Mann et al. in turn, found a substantial reduction of MPVCs in patients with the *SCN5A* R222Q mutation by known sodium channel blocking agents, such as amiodarone or flecainide [14].

Our data support the spectacular results by Laurent et al. [10] of the quinidine treatment in another family with DCM and MEPPCs. The standard heart failure therapy in our patient and his children was ineffective. In younger generation, it led directly to HTx (son) and the death of the patient’s daugther. We observed a significant reduction of MPVCs during the quinidine treatment (reduction MPVCs > 80%/day to 3-5% /day) and a significant improvement of the LV function. The earlier treatment with amiodarone was partially successful but had to be stopped due to its toxicity. Of note, the quinidine treatment was effective in the patient who also had co-existing CAD.

The effects of quinidine were tested on the cellular model [10]. Clinically, quinidine primarily works by blocking the fast inward sodium current (I_Na_) and the transient outward current (I_to_), by repolarising the delayed rectifier current I_Kr_, and by inhibiting Ca^2+^ and other K^+^ currents [18]. Quinidine usage caused the normalisation of Purkinje and ventricular cells potentials (maximal drug dose, remaining 50% I_Na_ and 30% I_Kr_ and I_to_). Such effect was still observed when the drug dosage was reduced (remaining 75% I_Na_ and 45% I_Kr_ and I_to)_, but disappeared in the case of a lower dosage of quinidine (remaining 85% I_Na_ and 50% I_Kr_ and I_to_) [10].

Current guidelines inform that Class Ia antiarrhythmic agents are contraindicated both in heart failure and in CAD. The ESC/AHA guidelines for the treatment of CHF or Ventricular Arrhythmias do not recommend class I drugs (quinidine, flecainid) to treat MPVCs in DCM patients [9, 19].

Current guidelines also advise genetic studies in patients with DCM and conduction disease [9]. Recent data show that patients with familial DCM and accompaning MEPPCs also need individual clinical diagnostics, including genetic examination toward Na_v_1.5 mutations. In such cases antiarrhythmic therapy containing sodium channel blockers is supposed to be successful. Only a limited number of published reports on families with the *SCN5A* R222Q Na_v_1.5 mutation, show a promising antiarrhythmic effect of the quinidine [10], flecainid and amiodarone therapy [14]. Case reports published up until now show that the reduction of MPVCs can partially or completely reverse LV dilation and reverse heart failure symptoms; however, the influence of the treatment on the prevention of sudden cardiac death is unknown. Our study adds to the existing literature with information that, the quinidine treatment was effective in the patient who also had co-existing CAD.

### Translation medicine

The mutations of the *SCN5A* gene in the DCM patients are an important cause of life-threatening ventricular arrhythmias. Based on the confirmed individual specific *SCN5A* mutation and pharmacokinetic experimental models of antiarrhythmic drugs, we can treat patients with well-matched sodium channel blockers like quinidine in our case.

Unfortunately, genetic testing is still difficult to obtain in general cardiologist practice. However, it gives perspective for future mutation-specific therapy dedicated for Purkinje cell related arrhythmia. Our case highlights the value of genotype information for the treatment strategy.

In conclusion, patients with familial DCM and MPVCs should be investigated for *SCN5A* gene mutations. The antiarrhythmic treatment with quinidine can significantly reduce the number of MPVCs and reverse LV dilation in a few months even in the presence of concomittant CAD.

## Abbreviations

ACEI: Angiotensin-converting enzyme inhibitor
AF: Atrial fibrillation
ASA: Acetylsalicylic acid
CAD: Coronary artery disease
DCM: Dilated cardiomyopathy
ECG: Electrocardiogram
ESC/AHA: European Society of Cardiology/American Heart Association
HF: Heart failure
HTx: Heart transplantation
ICD: Implantable cardioverter-defibrillator
LAD: Left anterior descending coronary artery
LBBB: Left bundle branch block
LQTS: Long QT syndrome
LV: Left ventricle
LVEF: Left ventricle ejection fraction
MEPPCs: Multifocal ectopic Purkinje-related premature contractions
MPVCs: Multifocal premature ventricular contractions
Na_v_1.5: Cardiac sodium channel alpha subunit
nsVT: Non-sustained ventricular tachycardia
NYHA: New York Heart Association
RBBB: Right bundle branch block
VT: Ventricular tachycardia
WES: Whole-exome sequencing
